# 2D Triangulation of Signals Source by Pole-Polar Geometric Models

**DOI:** 10.3390/s19051020

**Published:** 2019-02-27

**Authors:** Aleksandro Montanha, Airton M. Polidorio, F. J. Dominguez-Mayo, María J. Escalona

**Affiliations:** 1 Web Engineering and Early Testing (IWT2) research group, Departamento de Lenguajes y Sistemas Informáticos, Escuela Técnica Superior de Ingeniería Informática, Universidad de Sevilla, Avda. Reina Mercedes s/n, 41012 Seville, Spain; 2 Departamento de Informática Centro de Tecnologia, Universidade Estadual de Maringá, Av. Colombo, 5790 - Jd. Universitário, Maringá 87020-900, Brazil; ampolidorio@uem.br; 3 Departamento de Lenguajes y Sistemas Informáticos, Escuela Técnica Superior de Ingeniería Informática, Universidad de Sevilla, Avda. Reina Mercedes s/n, 41012 Seville, Spain; fjdominguez@us.es (F.J.D.-M.); mjescalona@us.es (M.J.E.)

**Keywords:** signal processing, 2D point location, computational geometry, pole-polar geometry

## Abstract

The 2D point location problem has applications in several areas, such as geographic information systems, navigation systems, motion planning, mapping, military strategy, location and tracking moves. We aim to present a new approach that expands upon current techniques and methods to locate the 2D position of a signal source sent by an emitter device. This new approach is based only on the geometric relationship between an emitter device and a system composed of m≥2 signal receiving devices. Current approaches applied to locate an emitter can be deterministic, statistical or machine-learning methods. We propose to perform this triangulation by geometric models that exploit elements of pole-polar geometry. For this purpose, we are presenting five geometric models to solve the point location problem: (1) based on centroid of points of pole-polar geometry, PPC; (2) based on convex hull region among pole-points, CHC; (3) based on centroid of points obtained by polar-lines intersections, PLI; (4) based on centroid of points obtained by tangent lines intersections, TLI; (5) based on centroid of points obtained by tangent lines intersections with minimal angles, MAI. The first one has computational cost O(n) and whereas has the computational cost O(nlogn) where n is the number of points of interest.

## 1. Introduction

Signals can be of various natures, including sound waves, visible and non-visible light, or other electromagnetic spectrum energies (radio waves or radar waves, for instance). The position of a signal emitter or receiver device can be estimated (located) by triangulating the signal data that it sends/acquires. The signal data comprise: the nature of the own signal (as light (electromagnetic energy), sound and vibration); its intrinsic attributes (such as power, frequency, and amplitude); its extrinsic attributes (as the time when the signal arrives at each particular receiver; the strength and the propagation angle of a signal acquired at each particular receiver). Current approaches used to locate an emitter or a receiver can be deterministic, statistical or machine-learning methods. Thus, we propose to perform this triangulation by geometric models that explore elements of pole-polar geometry. Here, we are presenting five geometric models to solve the point location problem: (1) based on centroid of a set of polar-points (PPC); (2) based on a convex hull region defined by a set of interest points (CHC); (3) based on centroid of a set of interest points obtained by polar-lines intersections (PLI); (4) based on centroid of a set of interest points obtained by tangent lines intersections, (TLI); (5) based on centroid of a set of interest points obtained by tangent lines intersections with minimal angles (MAI). 

The first one has computational cost O(n), whereas the others cost O(nlogn). The n value is the number of points of interest. 

For a system composed of m receivers, the maximum of polar-points of interest generated is $p=\left(\begin{array}{c} m\\ 2 \end{array}\right)=m!/\left(2\left(m-2\right)!\right)$. For PPC, CHC and PLI models n=p. For TLI and MAI models n=2p. In terms of m, the cost of proposed methods is $O\left(m^{2}\right)$ in accordance with the cost to perform $\left(\begin{array}{c} m\\ 2 \end{array}\right)$ combinations.

We use IEEE 802.11 Network infrastructures to evaluate the precision of these geometric models, with the aim to acquire strength signal data from emitter-receiver system. The acquired data was not preprocessed. The acquired raw data are used to provide better analysis of the errors committed by the five geometric methods proposed in this work (PPC, CHC, PLI, TLI, and MAI) in comparisons with the errors committed by Newton–Rapson (NRm), Least-Square (LSm), and Weighted Least Squares (WLSm) which have computational cost $\sim O\left(m^{3}\right)$. This paper does not address operations or methods related with signal data preprocessing. If you have particular interest in these operations, see Section 3.2 in this work. This means that there are errors in the acquired data used in this work. Such errors are due to the signal multipath; the presence of obstacles and the co-presence of other electromagnetic sources (see Section 1.1). The innovation proposed in this work is to use geometric methods to solve a geometric problem (estimation of the location of signal source or a receiver device). Obviously, in order to guarantee the accuracy of a location, it is necessary to ensure data with high quality.

Signal location estimation by range-based methods needs to solve a nonlinear equations system (quadratic equations). To facilitate this solution, the equations of this system are linearized by subtracting equations, that is, any equation of this system is chosen and is used to subtract all other ones from this system, and thus all terms of all equations that have degree two are eliminated from these equations and the system becomes linear. However, the realization of this subtraction also eliminates from the system the equation chosen to perform this linearization. For example, if there were five equations before the linearization, after the linearization there will be four equations in the system. It is important to consider that this equation that was eliminated contemplates data of radial range of the signal acquired by some device of the location network. Eliminating this equation means eliminating this device from this network and its participation on the final result of the location of a point in space becomes indirect. The most complicated fact of performing the linearization of the equations system by subtraction of its equations is in the possibility of propagation of the errors. Assume that the equation chosen to perform the linearization operation is that corresponding to the device that acquired the signal data with the highest error. By using this equation to subtract all other ones, such errors are seriously propagated to the linearized equations system, with impairment for the accuracy of the solution.

In this work we propose five geometric methods capable of estimating the location of a point (2D) without the requirement to solve a nonlinear equations system. This means that all signal radial range data acquired by all devices belonging to a positioning network are used directly to estimate the location of a desired point with computational cost $O\left(m^{2}\right)$ without propagating data errors among equations.

### 1.1. Considerations about Signal Data

In this section we briefly describe about the signal data acquisition and limitations, including the restrictive conditions involved in this acquisition. We try to associate the acquisition of data with the usual methods applied to the spatial location of a point of interest.

What locates what? There are applications which needed estimating the location of an emitter device (e.g., an airplane black box). There are applications which a receiver device needs to be located (e.g., a GPS unit). All methods presented in this paper are able to operate in any of these applications. The experimental cases (Section 3) adopt the convention for locating a receiver device.

The standard techniques range-based applied to location estimation uses a server to that aim. They perform the localization in two steps: (1) ranging, where the radial distance between two sensors (one with known positions and another with unknown position) is obtained, by some methods such as TOA (Time of Arrival), TDOA (Time Difference of Arrival), RSSI (Received Signal Strength Indicator) or AOA (Angle of Arrival) and (2) location computation, where the unknown location of a device is calculated by estimation through methods such as triangulation using the radial distance or angle data [1].

A Received Signal Strength Indicator (RSSI) is a measure of the power contained in a propagated signal. In an IEEE 802.11 system, RSSI refers to received radio signal strength (power level) in a wireless environment, measured in arbitrary units, that a particular signal is received by a receiver. Therefore, the higher the RSSI value is, the stronger the signal is received, and closer are the emitter and the receiver. RSSI-based techniques require the value of the signal magnitude attenuation to find the radial distance among a set of receiver devices. It is a practical and self-organized method that utilizes more power to send a large influx of data to the central server.

Received Signal Strength (RSS)-based fingerprinting approaches have been widely exploited for mobile device localization. The received signal strength is a function of radial distance between emitter and receiver devices. The strength magnitude of a received signal can vary because there are fonts of interferences (noise) in the signal propagation path, especially in outdoor environments [2]. Reference [3] explored signal strength data to feed a Perceptron and Decision Systems based on Learning Vector Quantization to obtain a fingerprint map of that radio strength signal. Reference [4] investigated the effects of hardware orientation, location, measurement time and duration, as well as the interference of the user presence on the RSS acquired data.

In the simplest case of free space propagation, electromagnetic waves radiate out of an isotropic antenna in all directions without being perturbed. The received power, any point in space, for such a case is provided by Friis equation [5,6] given by (1).

(1) $$\rho_{r}=\rho_{e}g_{e}g_{r}{\left(\frac{\lambda }{4\pi d}\right)}^{2},$$

$\rho_{r}$ and $\rho_{e}$ are the receiver and the emitter power
$g_{r}$ and $g_{e}$ are the receiver and emitter antennas gains
d is the radial distance between an emitter and a receiver in meters.
λ is the wavelength. For IEEE 802.11b and g to 2.4 GHz, λ≈0.125 m and, to 5.7 GHz, λ≈0.06 m.

The wave propagation of Electromagnetic Radiation (EMR) in the Earth’s free space conditions is compromised primarily by three phenomena [7]: (1) reflection. It happens when EMR interacts with a smooth surface with larger dimension than the wavelength; (2) diffraction, or shadowing. It takes place when the wave is obstructed by objects larger than the wavelength (a secondary wave is created behind the object); and (3) scattering. It occurs when the waves encounter a rough surface whose dimensions are of the same order as the wavelength [6]. As the wireless signal radiates out of the emitter antenna, it may interact with reflective surfaces and be reflected in different directions. If a fraction of this reflected signal reaches the receiver antenna, it will acquire signals from the same emitter that traveled by different paths. The reflected signal may be out of phase with the Line-Of-Sight (LOS) signal that can result in destructive interference and fade the receiver signal. The received signal is affected by different environment components (e.g., the own air) that promote the signal attenuation.

If the signal does not suffer external interference, it is expected an inverse exponential relationship between the radial distance d that the emitter device is there from the position of an AP and the value RSSI ρ of this signal measured in that AP [8]. In the dry air, the signal attenuation A is given by (2).

(2) $$A=20{log}_{10}\left(\frac{4\pi d}{\lambda }\right)$$

Therefore, for a signal propagation in an environment consisting of dry air, we can argue that the signal strength $\rho_{r}$ received by the receiver r is dependent of the signal strength $\rho_{e}$ that is produced by the emitter e, of the gains $g_{r}$ and $g_{e}$ promoted by sensors antennas and of the attenuation A, (3), promoted by the dry air.

(3) $$\rho_{r}=\rho_{e}+\rho_{g}-A$$

RSSI is used as a metric in most of distance measurement algorithms that run in indoor environments. Nevertheless, at a more basic level the reliability and precision of using RSSI to determine a spatial position in an outdoor environment has not been extensively experimented and there can be interference caused by structures. This monotonous behavior between the radial distance d and the signal strength ρ is not always observed.

Time of Flight (TOF) are time-based methods for measuring distance using signals data that propagate in a given environment with a known speed v. They consist in measuring the time interval that a wave (e.g., light, radio, or sound) needs to travel a spatial distance. Measuring radio or light wave propagations across small distance requires a clock with subnanosecond time measure; for sound it must be measured by millisecond. TOF-based methods are Time of Arrival (TOA), Time Differential of Arrival (TDOA), and Time of Transfer (TOT).

The emitter and the receiver clocks must be perfectly synchronized for using TOA approach. When an emitter sends a signal at the instant $t_{E}$, the receiver acquire this signal at instant $t_{R}>t_{E}$, so, the time interval spent for the signal arrival from emitter to receiver, $\Delta t=t_{R}-t_{E}$, allows computing the radial distance d that separates the emitter from the receiver by d=vΔt. In the 2D case, knowing the emitter distance from two receivers is sufficient to perform the triangulation of emitter location estimation. The TOT method is an unsynchronized-time approach that computes the radial distance between emitter and receivers based on the time delay of a standardized signal ξ (message) exchanged between the emitter and those receivers.

Accuracy of time acquisition is very important for any type of TOF measurements. An EMR wave travels 1 m in 3 ns in air, thus if a locating system with 1m of accuracy is desired, a 300 MHz clock (least) must be used for time acquisition. Clock accuracy depends on clock granularity, which is associated with the size of the smallest time interval measured by a clock. Larger granularity implies a small time interval, then greater precision [9]. A granularity of 20 ppm has an inaccuracy of 1.2 ms per minute, a rubidium clock has an inaccuracy of 0.06 ns per minute, and a caesium clock has an inaccuracy of 6 ps per minute. To sum up, clock precision is a source of error in TOF methods. The sources of errors can be intrinsic (i.e., related to the hardware) or extrinsic (i.e., related to the environment). Intrinsic errors are related to: (1) clock measurements accuracy; (2) printed circuit board trace (circuit impedance), that means that the electronic signal travels some distance on a printed circuit board trace and this internal path of the signal causes delay on signal propagation; (3) difference in the phases between emitter and receiver; and (4) relative velocity on signal treatment between emitter and receiver, which promotes frequency errors and, hence clock errors. Extrinsic errors comprise: (1) signal reflection; (2) diffraction; (3) scattering; (4) absorption/attenuation; (5) environmentally-dependent the speed signal propagation; and (6) physical model and computational errors (truncation and rounding). Reference [6] outlined sources of error and considerations about some methods of localization using radio frequency.

TOF-based methods suggest inaccuracy does not increase when distance increase. In general, for methods based on RSSI and angle AOA the error increases when distance increase.

To apply the TDOA approach, the clocks of the receivers system or of the emitters system need to be synchronized (e.g., for GPS, only the clocks of the satellites constellation need synchronized—this means that a mobile device GPS unit is not an expensive device). However, each satellite in this constellation needs to send in its signal the coordinates of its spatial position and the precise time registered by its clock. According [7], to estimate a position of a point using a satellite navigation system, it is thus necessary to have two things ready: the position of the satellites and the distance of the point from these satellites. Range observation equations are generated from this information and the position is estimated in 3D space by solving these equations. Therefore, two aspects are important at this point: to obtain this required range data and to solve the equations

### 1.2. Standard Methods and Considerations

#### 1.2.1. RSSI Approach 

The first system used for tracking and locating a mobile device by radio frequency in an indoor environment was RADAR [10]. Previously, [11,12] used the infrared to locate devices. Nevertheless, there are many limitations in the latter when using this range of the electromagnetic spectrum energy to establish communication among sensors and limited results were obtained. In contrast, the RADAR system operates by processing signal strength data at multiple base stations (AP, Access Point) geometrically positioned to provide overlapping coverage in the area of interest. The system enables estimating the location of an emitter by applying empirical measurements with signal propagation modeling.

The empirical models proposed by Equations (4) and (5) present significant disparities when confronted by actual data due to signal attenuation promoted by the floor and walls of an indoor environment. Reference [10] observed that sampled data could be filtered to achieve better results. Considering data median, model (5) provides a precision of 4.3 m, compared to 2.94 m for model (4) and 8.16 m for the strongest signal data values. For the 25th percentile, model (5) provides a precision of 1.86 m, whereas model (4) provides 1.92 m and 4.94 m for the strongest values. To summarize, this evaluation [10] shows that signals can suffer from low and high frequencies interferences (noise). Besides, [10] performed experiments to determine the impact of the data sample size on the result precision. He shows that using two data values by sample, the accuracy worsens by 11%, while using three data values it worsens by 4% (the larger the sample is, the more accurate the result is).

(4) $$\rho \left(d\right)=\rho_{0}-10ℒ{log}_{10}\left(\frac{d}{d_{0}}\right)-w\left(nW,C\right),$$

Or

(5) $$\rho \left(d\right)=\rho_{0}-20{log}_{10}\left(\frac{4\pi d}{\lambda }\right)+x_{\sigma },$$

where,

ρ(d) is the signal strength value (dB) expected by an AP located at a radial distance
d from the signal origin.
$\rho_{0}$ is the signal strength value (dB) at some reference distance $d_{0}$.
ℒ indicates the rate at which the path loss increases with distance (empirical value).
w(nW,C) is the signal attenuation factor promoted by walls.
λ is the signal wavelength.
$x_{\sigma }$ is a Gaussian noise with zero-mean and variance $\sigma^{2}$.

Considering w(nW,C)=0, in free obstructions outdoor environment, and measure $\rho_{0}$ at $d_{0}=1\;m$ from the AP, by (4), the radial distance d can be computed by (6).

(6) $$d=10^{\frac{\rho_{0}-\rho \left(d\right)}{10ℒ}}.$$

The parameter path-loss exponent ℒ (6) depends on the frequency of signal and environment interferences (e.g., walls, buildings, cars, rain, air moisture, or people); for open free space, ℒ≅2, and for environments with obstructions, ℒ>2. A more appropriate ℒ value can be determined in the system calibration phase [13]. Experiments carried out by [14] showed that in an urban area, the path loss variation ranges 2.7≤ℒ≤3.5, using a cellular radio.

#### 1.2.2. Time-Based Approaches (ToA and TDoA)

Reference [15] use sound waves to locate a receiver device using signal data based on TDoA measurements. The location computation is formulated as an optimization problem applying a set of constraint previously determined. The basic idea is to shrink the radius of all circles at a constant step length until the intersection area reaches a small threshold. This operation needs to perform N acquisitions of data (called phase). For each set of acquired data is applied an optimization algorithm. These acquisitions of data end when the optimization algorithm finds a solution considered satisfactory. The computational complexity of the proposed method is about $O\left(10\left(nN^{2}+n^{2}N\right)\right)$, where n is the number of nodes (emitters). 

Time-based approaches are based on electromagnetic (or sound) wave propagation that moves at a speed v close to light speed, $c\approx 3\times 10^{8}\;m/s$ (in the vacuum). In the Earth’s atmosphere v=c/n, n is the refraction coefficient of the atmosphere (n=1.000292 to dry air at conditions 0 °C and 1 atm. The accurate value is v=299,704,944  m/s). The physical model (7) responsible for computing the radial distance d between emitter and receiver depends only on the instant $t_{E}$ when the emitter sends the signal and the instant $t_{R}$ when the receiver get it.

(7) $$d=v\left(t_{R}-t_{E}\right)$$

The model (7) can be applied only in case that emitter and receiver are time-synchronized. Ensuring this synchronization is not always a simple task. It must be considered that v is an astronomical quantity and small deviations of precision in $\left(t_{R}-t_{E}\right)$ results in large errors (for each 1 ms error in the time registration there can be a location error higher than 300 km).

#### 1.2.3. Generalization of 2D Location Function Range-Based

In 2D space (x,y) are two unknowns for the location of a point P. In Cartesian coordinates, the point (x,y) can be represented in terms of its range by ${\left(x-x_{1}\right)}^{2}+{\left(y-y_{1}\right)}^{2}={\left(r_{1}\right)}^{2}$, where $\left(x_{1},y_{1}\right)$ is a known reference point and $r_{1}$ is the range (or radial distance) between the unknown point (x,y) and the reference point (x,y). To solve (x,y) is necessary a second observation given by another reference point $\left(x_{2},y_{2}\right)$ that generates the new circle equation ${\left(x-x_{2}\right)}^{2}+{\left(y-y_{2}\right)}^{2}={\left(r_{2}\right)}^{2}$. There are two solutions for these two quadratic equations. If these two solutions are the same, then these two circles are tangent and the solution for P is found. If these two circles are secant, *P* has two ambiguous solutions. In this case, to obtain the correct solution is necessary known a third reference point $\left(x_{3},y_{3}\right)$ and its respective $r_{3}$ range to P. This is known as trilateration, the estimation of the position of a point unambiguously based on the measurements of distances from three or more known reference locations [7]. Otherwise, these three circles do not intercept at a single point because there are errors in ranges measurements. In this case, the solution for P is inaccurate and need to be estimated with error minimization. To provide this error minimization it is necessary known others reference points $\left(x_{k},y_{k}\right)$ and its respective ranges $r_{k}$—see Equation (8). As the Cartesian plane is concerned, the Euclidean distance between two points $P_{0}\left(x_{0},y_{0}\right)$ and $P_{1}\left(x_{1},y_{1}\right)$ is given by $d=\sqrt{{\left(x_{1}-x_{0}\right)}^{2}+{\left(y_{1}-y_{0}\right)}^{2}}$, so, by means of (6) or (7), the relationship ${\left(x_{1}-x_{0}\right)}^{2}+{\left(y_{1}-y_{0}\right)}^{2}={\left(v\left(t_{R}-t_{E}\right)\right)}^{2}=10^{2\frac{\rho_{0}-\rho \left(d\right)}{10ℒ}}$ is obtained, which is the circle equation with radius $r=v\left(t_{R}-t_{E}\right)=10^{\frac{\rho_{0}-\rho \left(d\right)}{10ℒ}}$ and center $\left(x_{0},y_{0}\right)$. If $P_{0}$ represent the receiver position and $P_{1}$ the emitter position, the emitter belongs (best case) to this circle line and the receiver is at the circle center. Note that signal-based location methods need to know the value of the radial range of the signal r regardless of whether this value is estimated by strength data or the wave propagation speed of that signal. Despite of how the value of r is estimated, such methods do not change. Therefore, performing experiments using signal strength and signal propagation velocity data is irrelevant to compare the accuracy achieved by different methods.

The radial distance (or range data) d=r between emitter and receiver is identified through (6) or (7). Using this concept to m>2 receivers by one emitter, the following is obtained (8)

(8) $$F\left(x,y\right)=\{\begin{array}{c} {\left(x-x_{1}\right)}^{2}+{\left(y-y_{1}\right)}^{2}-{\left(r_{1}\right)}^{2}=0\\ {\left(x-x_{2}\right)}^{2}+{\left(y-y_{2}\right)}^{2}-{\left(r_{2}\right)}^{2}=0\\ \begin{array}{c} \vdots \\ {\left(x-x_{m}\right)}^{2}+{\left(y-y_{m}\right)}^{2}-{\left(r_{m}\right)}^{2}=0 \end{array} \end{array}$$

**Figure 1:**
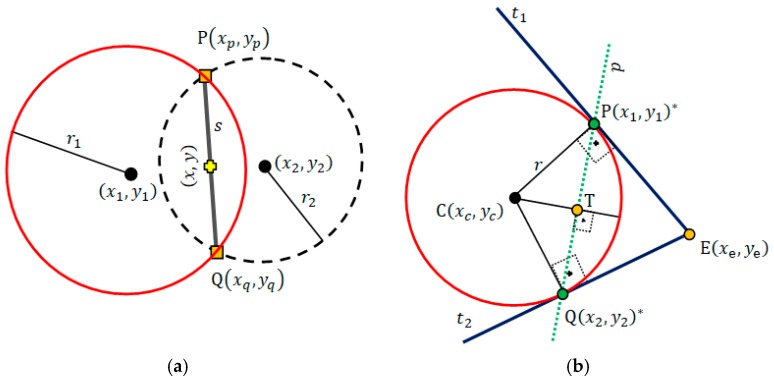
(**a**) Analytical solution to (x,y) when there is an intersection between circles. (**b**) Possible tangential straight lines $t_{1}$ and $t_{2}$ for the circle line obtained by an external point $E\left(x_{e},y_{e}\right)$ (pole-point). Polar-line p and its geometric relationship with the tangent lines and a given conic section (circle in this case).

The Equation (8) is a nonlinear equations system to solve the emitter location (x,y). If m=2, this equations system is normal and can be solved either by a numerical or empirical method or applying the analytical solution showed in Figure 1a. If m>2, the system is redundant and allows minimizing the solution error by means of Nonlinear Least-Squares (NLLS). 

#### 1.2.4. How to Solve the System

The following are some possible solutions for the system of nonlinear equations given in (8). Solution by Least-Squares Method—For this solution [16] proposes a linearization of the non-linear equation system (8). This linearization is achieved when an equation of the system (8) is used to subtract all the others. By performing this subtraction, the system (8) with non-linear equations results in a system of m−1 linear equations. In this case, to solve a 2D location problem, m≥3 receivers are necessary. For example, if the equation 1 of (8) is chosen to perform this linearization, then the resulting linear system, written in the form $M{\left[x,y\right]}^{t}=b$, is given by (9). The solution of $M{\left[x,y\right]}^{t}=b$ is given by ${\left[x,y\right]}^{t}=M^{-1}b$, if m=3, or by ${\left[x,y\right]}^{t}={\left(M^{t}M\right)}^{-1}M^{t}b$, if m>3.

(9) $$M={\left[\begin{array}{cc} x_{2}-x_{1} & y_{2}-y_{1}\\ x_{3}-x_{1} & y_{3}-y_{1}\\ x_{4}-x_{1} & y_{4}-y_{1}\\ \vdots & \vdots \\ x_{m}-x_{1} & y_{m}-y_{1} \end{array}\right]}_{m-1\times 2}\;\mathrm{and}\;b=\frac{1}{2}{\left[\begin{array}{c} b_{2}-b_{1}\\ b_{3}-b_{1}\\ b_{4}-b_{1}\\ \vdots \\ b_{m}-b_{1} \end{array}\right]}_{m-1\times 1}$$

where $b_{k}=r_{k}^{2}-\left(x_{k}^{2}+y_{k}^{2}\right)$, for k=1,2,3,…,m.

Note that these solutions have any mechanism to handle errors involved with the acquired data. The position solution obtained by (9) was based upon a fundamental assumption that the ranges have no error in them. However, the same approach is valid when the range errors for all the reference points are statistically independent and identical. However, this is not true in real cases. Often, this error is neither independent nor identical. Under such conditions, the least squares position estimate does not hold good as an optimal estimate. If the range errors are Gaussian and the covariance of ranging errors is given by the matrix Σ, then the optimal estimation for location is given by the Weighted Least Squares (WLSm) provide by ${\left[x,y\right]}^{t}={\left(M^{t}\Sigma^{-1}M\right)}^{-1}\left(M^{t}\Sigma^{-1}b\right)$ [7,16,17].

Solution by Numerical Method—For this specific case, the Jacobian matrix J (10) is the matrix of first order partial derivatives of F(x,y), such that, J(F(x,y)) is given by

$$J\left(F\left(x,y\right)\right)=\left[\begin{array}{cc} \frac{\partial f_{0}}{\partial x} & \frac{\partial f_{0}}{\partial y}\\ \frac{\partial f_{1}}{\partial x} & \frac{\partial f_{1}}{\partial y}\\ \vdots & \vdots \\ \frac{\partial f_{m-1}}{\partial x} & \frac{\partial f_{m-1}}{\partial y} \end{array}\right],$$

applying in (8)

(10) $$J\left(F\left(x,y\right)\right)=2\left[\begin{array}{cc} x-x_{0} & y-y_{0}\\ x-x_{1} & y-y_{1}\\ \vdots & \vdots \\ x-x_{m-1} & y-y_{m-1} \end{array}\right]$$

We look for F(x,y)=0. For m=2, an interactive solution for F(x,y)=0 is found by Newton’s method [18], by way (11)

(11) $$\left[\begin{array}{c} x_{k+1}\\ y_{k+1} \end{array}\right]{}_{2\times 1}=\left[\begin{array}{c} x_{k}\\ y_{k} \end{array}\right]{}_{2\times 1}-{\left[J(x_{k},y_{k})\right]}_{2\times 2}^{-1}{\left[F(x_{k},y_{k})\right]}_{2\times 1}$$

where k=0,1,2,…. is $k^{th}$ iteration, ${\left[F\left(x_{k},y_{k}\right)\right]}_{m\times 1}$ is vector function, ${\left[J\left(x_{k},y_{k}\right)\right]}_{2\times m}^{-1}$ is the inverse of the Jacobian matrix, and $\left(x_{0},y_{0}\right)$ is the first estimative for the solution. Such solution is found when ${\left[x_{k+1},y_{k+1}\right]}^{t}\leq {\left[\varepsilon ,\varepsilon \right]}^{t}$, and ε stands for the precision value specified a priori. If m>2 and $X_{k}={\left[x_{k},y_{k}\right]}^{t}$, the system (8) can be solved by (12).

(12) $$X_{k+1}=X_{k}-F\left(X_{k}\right){\left({\left[J\left(X_{k}\right)\right]}_{}^{t}\left[J\left(X_{k}\right)\right]\right)}_{}^{-1}{\left[J\left(X_{k}\right)\right]}_{}^{t}$$

Algebraic Solution—An algebraic 3D solution to locate a receiver ([19] aimed to locate a receiver using TDOA measurements and four satellites emitters) was found out by [19], Bancroft’s method, which reduces the problem to solve a quadratic equation and yields the 3D Cartesian coordinate of the receiver as well as the common time of signal transmission. It also performs several algebraic manipulations to reduce the equations to a least-squares problem. The presented solution (13) considers that

(13) $${\tilde{r}}_{i}^{2}={\left(r_{i}-\xi_{i}\right)}^{2}={\left\|p_{i}-\rho \right\|}^{2}={\left(x-x_{i}\right)}^{2}+{\left(y-y_{i}\right)}^{2}+{\left(z-z_{i}\right)}^{2},$$

where, $r_{i}$ and ${\tilde{r}}_{i}$ are the observed and actual radial distances (pseudorange and actual range, respectively); $\xi_{i}$ is the measurement noise at the receiver corresponding to the measurement between the $i^{th}$ satellite and the receiver, respectively and ρ is the receiver position (x,y,z) to determine. A generally acceptable modeling of the ranging error $\xi_{i}$ was described in [20]. Thus, using the receiver clock bias $–b=\xi_{i}$ as the only noise measurement and applying some algebra the following equation is obtained

$$\left(x_{i}^{2}+y_{i}^{2}+z_{i}^{2}-r_{i}^{2}\right)-2\left(x_{i}x+y_{i}y+z_{i}z-r_{i}b\right)+\left(x^{2}+y^{2}+z^{2}-r^{2}\right)=0.$$

Let $\rho ={\left[xyzr\right]}^{t}$ denotes the receiver position vector and $p_{i}={\left[x_{i}y_{i}z_{i}r_{i}\right]}^{t}$ denotes the satellite position and range vectors. According to Lorentz inner product for 4-space (given by $\langle \vec{u},\vec{v}\rangle =u_{1}v_{1}+u_{2}v_{2}+u_{3}v_{3}-u_{4}v_{4}$), can be rewritten as

$$\frac{1}{2}\langle p_{i},p_{i}\rangle -\langle p_{i},\rho \rangle +\frac{1}{2}\langle \rho ,\rho \rangle =0.$$

Now, the solution can be found utilizing least-squares estimation to the organized satellites equations in the matrix $B_{m\times 4}=\left[x_{k}y_{k}z_{k}-r_{k}\right],k=1\ldots m$ and the vector $a_{m\times 1}=\frac{1}{2}\left[\langle p_{k},p_{k}\rangle \right]$. The final solution demands a consistency analysis of the partial results found.

Analytical Solution—When two circles with centers at $\left(x_{1},y_{1}\right)$ and $\left(x_{2},y_{2}\right)$ and radius $r_{1}$ and $r_{2}$ intersect at one or two points $P\left(x_{p},y_{p}\right)$ and $Q\left(x_{q},y_{q}\right)$. This intersection do not always occurs due to noise and underestimation of ranges [21]. The analytical solution (x,y) is found at the median of the straight line segment s that connects P and Q points (Figure 1a), given by (14)

(14) $$\left(x,y\right)=\left(\frac{x_{p}+x_{q}}{2},\frac{y_{p}+y_{q}}{2}\right),$$

where

$x_{p}=\frac{-E+G}{H};\;x_{q}=\frac{-E-G}{H};\;y_{p}=\frac{A-Bx_{p}}{C};\;y_{q}=\frac{A-Bx_{q}}{C}$;
$A=r_{1}{}^{2}-r_{2}{}^{2}-x_{1}{}^{2}-y_{1}{}^{2}+x_{2}{}^{2}+y_{2}{}^{2}$; $B=-2\left(x_{1}-x_{2}\right)$; $C=-2\left(y_{1}-y_{2}\right)$; $L=\frac{B}{C}$; $D=1+L^{2}$;
$E=2\left(L\left[1-A\right]-x_{1}\right)$; $F=L\left(L-2y_{1}\right)+x_{1}{}^{2}+y_{1}{}^{2}-r_{1}{}^{2};\;G=\sqrt{E^{2}-4DF}$; H=2L.

For a normal equation system another analytical/algebraic solution for (8) can be obtained performing the linearization by row (equations) subtraction and then applying the Gauss–Jordan elimination method [18].

Despite the method chosen to solve this problem (analytical, algebraic, least-squares, numerical or NLLS), there are invariable errors associated with the acquired data. These errors are propagated by computational arithmetical operators, mainly multiplication and division.

It is necessary to minimize the number of arithmetic operations involved in the solution in order to improve the accuracy of the results and, therefore, reduce the computational cost to provide facilities for designing of real time systems. This work proposes a geometry-based solution with the aim to minimize the number of arithmetical operations so as to find the desired solution.

### 1.3. Useful 2D Geometric Definitions

This subsection describes some geometric important definitions for understanding the proposed geometric models. Such definitions are labeled as, for example, (d-9) which means the 9th definition and its uses are thus referenced in the text. References [22,23] provide these definitions and further information in this regard.

Point and Line—A coordinate P(x,y) define the point P in the Cartesian plane $\left({\mathbb{R}}^{2}\right)$. The line that passes through the points $P\left(x_{1},y_{1}\right)$ and $Q\left(x_{2},y_{2}\right)$, P≠Q, is denoted $\overset{↔}{PQ}$. The line segment $\bar{PQ}$ (with endpoints P and Q) is the portion of the line $\overset{↔}{PQ}$ between points P and Q.

**Figure 2:**
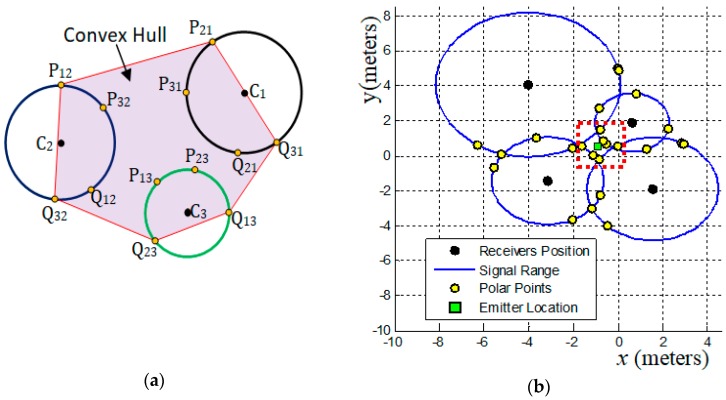
(**a**) Illustration of Convex Hull algorithm applied to a set of polar-points. (**b**) An example of signal data and it respective signal ranges with its polar points. This case represents sets of signal data with satisfactory quality. The red dotted rectangle mark a region where a set of polar points enveloping the location of an emitter device.

**(d-1)**  The Euclidean distance $D_{\bar{PQ}}$ between two points $P\left(x_{1},y_{1}\right)$ and $Q\left(x_{2},y_{2}\right)$ is given by
  $$D_{\bar{PQ}}=\sqrt{{\left(x_{1}-x_{2}\right)}^{2}+{\left(y_{1}-y_{2}\right)}^{2}}.$$
**(d-2)**  For constants A, B, C (A and B not both zero) all points (x,y) satisfying the equation
  Ax+By+C=0 define the implicit line equation in the Cartesian plane. For two points $P\left(x_{1},y_{1}\right)$ and $Q\left(x_{2},y_{2}\right)$, a particular $\overset{↔}{PQ}$ line equation is obtained by
  $$A=y_{1}-y_{2};\;B=x_{2}-x_{1};\;C=-Ax_{1}-By_{1}.$$
**(d-3)**  Two particular lines $A_{1}x+B_{1}y+C_{1}=0$ and $A_{2}x+B_{2}y+C_{2}=0$ have an interception at the point $\left(x_{+},y_{+}\right)$, if $d=A_{1}B_{2}-A_{2}B_{1}\neq 0$, given by
  $$\left(x_{+},y_{+}\right)=\left(\frac{B_{1}C_{2}-B_{2}C_{1}}{d},\frac{A_{2}C_{1}-A_{1}C_{2}}{d}\right),$$
  if $A_{1}A_{2}+B_{1}B_{2}=0$, lines are perpendicular. If $A_{1}B_{2}=A_{2}B_{1}$, lines are parallel or coincident.
**(d-4)**  The angle θ formed between two particular lines is given by
  $$\tan\theta =\frac{A_{1}B_{2}-A_{2}B_{1}}{A_{1}A_{2}+B_{1}B_{2}}.$$
**(d-5)**  The line equation $A_{p}x+B_{p}y+C_{p}=0$ that passes through point $P\left(x_{p},y_{p}\right)$ and is perpendicular to the line Ax+By+C=0 is defined as
  $$A_{p}=-B;\;B_{p}=A;\;\;C_{p}=Ax_{p}-By_{p}.$$
  Circle—In the Cartesian plane the equation ${\left(x-x_{c}\right)}^{2}+{\left(y-y_{c}\right)}^{2}=r^{2}$ defines the implicit circle equation centered at the point $C\left(x_{c},y_{c}\right)$ with radius r. Let $E\left(x_{e},y_{e}\right)$ be an external point to a circle. By using E we can obtain two tangent lines, $t_{1}$ and $t_{2}$, to the circle (Figure 1b), which pass through points $P{\left(x_{1},y_{1}\right)}^{*}$ and $Q{\left(x_{2},y_{2}\right)}^{*}$, respectively. Points P and Q can be computed by applying the geometric concept of pole-polar definition.
  Pole-Polar Geometry—Pole-point and polar-line are, respectively, a point and a line that have a unique reciprocal relationship with respect to a given conic section. If the point lies on the conic section, its polar-line is the tangent line to the conic section at that point [24]. If the pole-point is external to the conic section, the polar-line intercepts the conic section exactly at the points that allow passing tangent lines from this pole-point (Figure 1b). Our interest is to pass two tangent lines, $t_{1}$ and $t_{2}$, through a circle centered at point $C\left(x_{c},y_{c}\right)$ with radius r. Moreover, these lines must pass through a known external point $E\left(x_{e},y_{e}\right)$ (or pole-point) to this circle (Figure 1b). We need to locate the coordinates of the polar-points $P{\left(x_{1},y_{1}\right)}^{*}$ and $Q{\left(x_{2},y_{2}\right)}^{*}$, which define the polar-line p and lies to the tangents lines $t_{1}$ and $t_{2}$. Additionally we must find the equation of the polar-line p(Ax+By+C=0).
**(d-6)**  The general equation of a conic in the Cartesian coordinate system is given by $a_{xx}x^{2}+2a_{xy}xy+a_{yy}y^{2}+2b_{x}x+2b_{y}y+w=0$. We need the equation of the polar-line p(Ax+By+C=0) that can be obtained by a known pole-point $E\left(x_{e},y_{e}\right)$. The required coefficients of the respective polar-line p is given by: $A=a_{xx}x_{e}+a_{xy}y_{e}+b_{x}$; $B=a_{xy}x_{e}+a_{yy}y_{e}+b_{y}$; $C=b_{x}x_{e}+b_{y}y_{e}+w$. In this work, the expected conic section is a circle. For the circle case, the following simplifications are helpful: $a_{xx}=1$; $a_{xy}=0$; $a_{yy}=1$; $b_{x}=-x_{c}$; $b_{y}=-y_{c}$ and $w=x_{c}^{2}+y_{c}^{2}-r^{2}$.
  The next step consists in placing points $P{\left(x_{2},y_{2}\right)}^{*}$ and $Q{\left(x_{2},y_{2}\right)}^{*}$, which are obtained by computing the intersection between the polar-line p and the circle line (Figure 1b). To compute the intersection of a line, Ax+By+C=0, with a circle, ${\left(x-x_{c}\right)}^{2}+{\left(y-y_{c}\right)}^{2}=r^{2}$, the following conditions must be considered: $d_{lc}=\frac{\left|Ax_{c}+By_{c}+C\right|}{\sqrt{A^{2}+B^{2}}}$ is the distance between the line and the circle center point $C\left(x_{c},y_{c}\right)$,
  ○ if $d_{lc}>r$, there is no intersection point;
  ○ if $d_{lc}=r$, the line is tangent to the circle and has one intersection point;
  ○ if $d_{lc}<r$, the line is secant to the circle and has two intersection points.
  The algebraic solution for this intersection is an equation of degree two. Another way to solve this intersection is applying some geometric relationships, as follows. To find the intersections points $P{\left(x_{1},y_{1}\right)}^{*}$ and $Q{\left(x_{2},y_{2}\right)}^{*}$, which are the polar-points, we have first to drop a perpendicular line (by d-5) from the center $C\left(x_{c},y_{c}\right)$ of the circle to the line p. Let $T\left(x_{t},y_{t}\right)$ be the intersection point and $\overset{↔}{CT}$ be the line that passes through C and T (Figure 1b).
  The equation of line p(Ax+By+C=0) is known (by d-6). Thus, the equation of line $\overset{↔}{CT}$ is $\overset{↔}{CT}\left(-Bx+Ay+Ax_{c}-By_{c}\right)$. This way, the point $T\left(x_{t},y_{t}\right)$ can be computed by intersection between p and $\overset{↔}{CT}$ lines (by d-3).
  $D_{\bar{CT}}$ represents the Euclidean distance between points C and T; $D_{\bar{PT}}$ refers to the distance between points P and T; $D_{\bar{QT}}$ stands for the distance between points Q and T; and $D_{\bar{CP}}=D_{\bar{CQ}}=r$ (by d-1).
  The triangles ΔCPT and ΔCQT are right-angled, and hence we prove that
  $$D_{\bar{CT}}^{2}+D_{\bar{PT}}^{2}=r^{2},$$
  and
  $$D_{\bar{CT}}^{2}+D_{\bar{QT}}^{2}=r^{2}.$$
**(d-7)**  So, $D_{\bar{QT}}=D_{\bar{PT}}=h=\sqrt{r^{2}-D_{\bar{CT}}^{2}}$; now, if we translate the point T by h units in both directions along line p, the points P and Q are determined as follows
  $${\left(x_{1},y_{1}\right)}^{*}=\left(x_{t}-\frac{Bh}{\sqrt{A^{2}+B^{2}}},y_{t}+\frac{Ah}{\sqrt{A^{2}+B^{2}}}\right),$$
  and, if h≠0
  $${\left(x_{2},y_{2}\right)}^{*}=\left(x_{t}+\frac{Bh}{\sqrt{A^{2}+B^{2}}},y_{t}-\frac{Ah}{\sqrt{A^{2}+B^{2}}}\right).$$
  The suggested geometric models that use the convex hull algorithm (CHC, PLI, TLI, and MAI) need to minimize the region of interest (ROI) and exclude bad points from final results. This minimization of ROI is obtained by obtaining a convex polygon defined on a set of previously computed points. A set S is convex if $\forall x,y\in S\Rightarrow \bar{xy}\subseteq S$. Any region (polygon) with a “dent” is not convex [24]. The convex hull of a set of points is the smallest convex set containing these points [24,25,26].
**(d-8)**  The convex hull algorithm is used in this work to specify a Region of Interest (ROI).
  To illustrate the use of the convex hull we must consider the existence of three receivers centered at coordinates $C_{1}\neq C_{2}\neq C_{3}$ (and these coordinates cannot all be collinear) that collect a signal from a point with distance $r_{1},r_{2}$ and $r_{3}$ (radial distance), respectively. $P_{kj}$ and $Q_{kj}$, k≠j, represents the polar-points that lie to the circle centered in $C_{j}$ that is obtained by the external point $C_{k}$ (center of another circle), as Figure 2a shows. Thus, the smallest convex polygon that contains all obtained polar-points is the convex hull for these points and this minimal polygon defines our ROI. This region in red-color lines is used to illustrate the ROI for the proposed geometric models presented below (always representing a convex hull to define a ROI).
**(d-9)**  The location estimation of the emitter, $E_{xy}\left(E_{x},E_{y}\right)$, is based on a set S that contains n points, (x,y), which are collected in a defined ROI. This location is given by the centroid point among all points in S, by
  $$E_{x}=\frac{\sum_{x\in S}x}{n},\;E_{y}=\frac{\sum_{y\in S}y}{n}$$

Section 2 presents the models (methods) proposed in this work. All examples illustrated in Section 2 are generated in a simulator system (MatLab code) that implements the respective proposed models. This simulator is available for download (www.coding2change.org/articles/GeometricModels.zip). The simulated cases presented in this work use systems composed by three, four, or five receivers, but the simulator is able to operate with any number (>1) of receivers.

The Figure 2b presenting a Cartesian system to locate points in meters. In all figures bellow that involves example of location problems the description of the axes (label) were omitted. The axes *x* and *y* represents the Cartesian system to locate a point in the plan. The coordinate values are free of linear unit (meters, feet, or geographic coordinates).

## 2. The Proposed Geometric Models

The proposed geometric models are applicable to m≥2 receivers and one emitter. A system comprises m receivers to triangulate the emitter location $E_{xy}\left(E_{x},E_{y}\right)$ by geometric relationship applying elements of pole-polar geometry.

Our algorithmic descriptions (and the simulator) use as input data the coordinates of each receiver position $C_{k}\left(x_{k},y_{k}\right)$, k=1,2,…,m and its respective signal range $r_{k}$. In real world, these signal ranges values are computed from acquired signal data by each receiver using, for example, a time-based or strength-based model. Only after can be applied a method capable to triangulate the signal emitter device location.

In this section we present the proposed geometric methods. To evaluate such methods, we developed an experiment, in an outdoor environment, capable of acquiring signal strength data (Section 3). In this environment we define a local coordinate system that allows controlling the geometry of the localization system and guarantees precision in the positioning of the emitters and the receiver. The analyses of the acquired data in this experiment, the results produced by the application of the proposed methods and comparisons with results obtained by other three methods are in Section 3.2 and Section 3.3.

### 2.1. Accurate Data, Exact Result

Before presenting the five proposed geometric models to solve the problem of locating a signal source, we present now the perfect solution, if the acquired signal data are accurate and if the spatial geometric arrangement among the receivers be symmetric along the *x*-axis and *y*-axis. This exact solution has complexity O(n).

All the geometric models proposed in this work are based on the calculation of polar points, which belong to a circle line (signal range). If it is certain that the data acquired from the signal are accurate, the solution for the location of the signal source is immediate. According to ISO 5725-1, the term “accuracy” is used to describe the closeness of a measurement to the actual value. 

**Figure 3:**
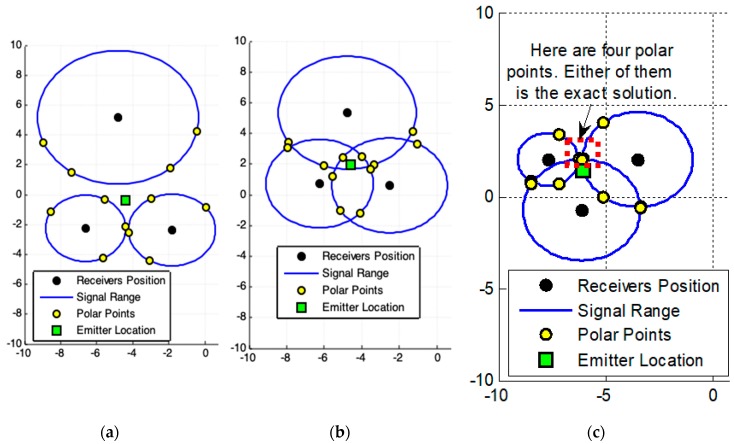
Results produced by Polar-Points Centroid Model (PPC). (**a**,**b**) show the possible presence of noise in acquired data. (**c**) Represents the best case for acquired data because the signal data are *quasi*-exact. The red dotted rectangle mark all (four) polar points that belong to all circle lines (signals range). The exact solution is one of these points (see Section 2.1).

**Figure 4:**
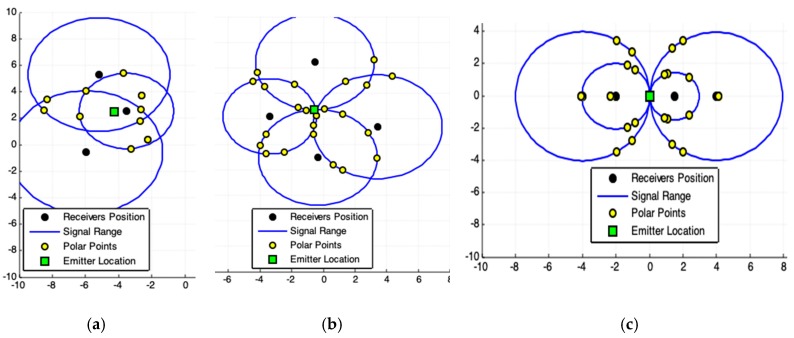
(**a**) Incoherence in data. The spatial position of a receiver is inside of range of another receiver. In this case, some polar-points are complex numbers (see the polar-points out of the line circles)—but their real parts belong to the region of interest (ROI). (**b**) Accurate data. Exact solution produced by PPC using redundant data (four receivers). For this case, is better applying the exact solution providing by Section 2.1. (**c**) Accurate data. A collinear arrangement among receivers (geometric arrangement not recommended) and the exact solution provided by PPC. The solution to this problem is impossible for Weighted Least Squares (WLSm), Least-Square (LSm), and Newton–Rapson (NRm).

The Figure 2b, Figure 3c, Figure 4b,c illustrate some cases that involve accurate signal data and therefore must have exact solutions. The Figure 3c is the perfect case using a system composed by m=3 receivers. The Figure 4b,c show perfect cases using a system with m=4 receivers. In fact, the case presented in Figure 3c is almost perfect. The signal data has been purposely shifted from perfection to being able to see that in the red dotted rectangle there is more than one polar point (there are four). If the data were accurate, these four polar points would self-overlap at the intersection point among the lines of these three circles. See in Figure 3c that the solution pointed out by the geometric model PPC (green rectangle) is not exact. The reason for this divergence is explained in the description and analysis of the PPC model (Section 2.2). For the case illustrated in Figure 3c, the exact solution is obtained by the set of polar points that overlap the point of intersection among the lines of the circles.

If the signal data are accurate, the exact solution is given by a single point, among all the polar points, that having the property to belongs at the same time to all circles lines (signal ranges). The formal proof for this exact solution is obtained by construction, or proof by example, which requires the construction of a concrete example with exact solution to show that some polar point is the exact solution because have the property to belongs to all circles lines. To verify this proof, use the proposed PPC method (Section 2.2) provided in the simulator system (see Section 4). Using the simulator, you can construct examples of ideal situations (all lines of circles must intersect at a single point and the geometric arrangement of the receivers in the plane must respect aspects of non-collinearity and neither all them can be located in the range of another receiver). If these conditions are met, the PPC method will determine one or more polar-points over that single point of intersection among all circle lines and/or determine the solution over that intersection.

To obtain the exact solution we must discover at least one polar point that has the property of belonging to all circles lines. The number of polar points that have this property depends on the geometric arrangement among the position of the receivers. For example, the arrangement presented in the Figure 3c has four polar points with this property. For Figure 4b, there are eight polar points. For Figure 4c, although the data is perfect, there is no polar point that has this property. Regardless of the number of receivers m used in the system, the maximum number of polar points that can have this property is eight.

The Figure 2b shows a system composed of four receivers. As you can see the lines of all circles do not intersect at a single point. In this case, there is some error associated with the signal data. Even so, we can admit that the data quality is satisfactory.

If the geometric arrangement among the position of the receivers is favorable and if the quality of the signal data is high there will always be an expressive set of polar points involving the location of the signal-emitting device. If the system consists of m≥3 receivers, find all polar points that are closest to each other. The centroid point (green rectangle in Figure 2b) among these selected points is the best solution to estimate the location of the signal emitter device. This can be done by stipulating a distance value as a threshold (acceptable error value) and verifying that each polar point is located a distance less than that threshold with respect to the lines of all circles (signal range). If there is a single circle line that is at a distance greater than the stipulated threshold from the point-candidate under analysis then that point must be discarded, otherwise it is selected.

However, receiving high signal data is an almost unlikely task. In this way, this work does not consider this eventual possibility as a solution. Our intention is to present new methods applied to signal triangulation problems and to analyze their behavior in the face of the errors inherent in the signal data.

### 2.2. Polar-Points Centroid Model (PPC)

The Figure 3 and Figure 4 show a set of possible cases of signal data and the respective application of PPC model (Algorithm 1). Figure 4a,c presents an intrinsic example of data incoherence, when the spatial position of a receiver is inside the signal range of another receiver, due to the presence of noise or excessive gain applied to the antennas. Even so, the results produced are consistent with data and no singularity was generated. If this case is possible, then some polar-points are complex numbers in the form a+bi. However, the proposed models have their origins in the polar geometry projection, which is also used to represent complex numbers. This means that the real part, real(a+bi)=a, of a complex polar-point can be used as an approximate value to represent an actual polar-point. Another way is to exclude all generated complex numbers from partial results. In this work, those complex numbers are not excluded. This option shows that the proposed geometric models are capable to solve these data incoherence producing a consistent result (Figure 4a,c, Figures 8b and 13).

Figure 3c displays the optimal case for real application. The emitter location should be in the common intersection point among the three receiver range circles. Although the result was close to this point, there is no perfect match, because there are polar-points that do not have the pattern responsible for symmetrically enveloping the emitter location. These polar-points are bad points. Using redundant data, by applying m>3 receivers to the system, these non-symmetrical points will have low influences on results (Figure 4b).

Figure 4c present an atypical geometric arrangement for a system used to solve signal triangulation problems. All receivers are arranged in a straight line (they are collinear). It is not all triangulation methods that can solve the problem with this arrangement. The PPC resolves and, if the data is accurate, the solution can be exact.

**Algorithm 1.** PPC—Polar-Points Centroid Model 
**Data Input**
m≥2 is the number of receivers. 
$C_{k}\left(x_{k},y_{k}\right)$, k=1,2,…,m, is the planar position of each receiver. 
$r_{k},k=1,2,\ldots ,m$, is the signal range of each receiver to an emitter.
**Procedure**
1: **for** each k=1,2,3,…,m
 2: ∀k,j:1,2,…,m/k≠j, by (d-6) and (d-7), for each receiver position $C_{k}$, used as pole-points, **compute** all combinations of polar-points $P_{kj}\left(x_{pkj},y_{pkj}\right)$ and $Q_{kj}\left(x_{qkj},y_{qkj}\right)$ with the respective receiver at position $C_{j}$ and signal range $r_{j}$.  3: **store** the points $P_{kj}$ and $Q_{kj}$ in the set $\left(S\leftarrow S\cup \left\{P_{kj},Q_{kj}\right\}\right)$.4: **end for**5: **Apply** (d-9) in the set S, compute the location estimation, $E_{xy}\left(E_{x},E_{y}\right)$, of the emitter.
**Information Output**
6: Emitter location estimation $E_{xy}\left(E_{x},E_{y}\right)$.

### 2.3. Convex Hull Centroid Model (CHC)

**Figure 5:**
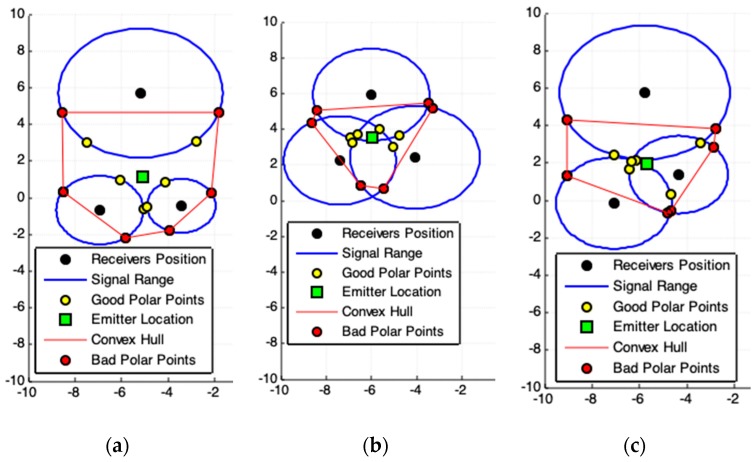
Results produced by Convex Hull Centroid Model (CHC). (**a**,**b**) highlight the possible presence of noise in acquired data. (**c**) Represents the best case for acquired data. For this case, is better applying the exact solution provided by Section 2.1.

**Figure 6:**
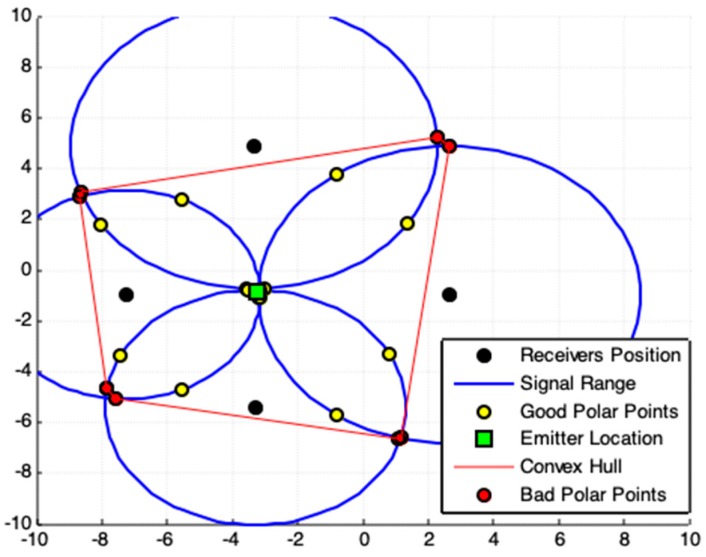
Results produced by CHC using satisfactory redundant data (four receivers). For this case, is better applying the exact solution provided by Section 2.1.

Figure 5a,b shows results closer to the solutions together with those shown in Figure 3a,b. Nonetheless, the result shown in Figure 6 is better than that of Figure 3c and Figure 5c. This improvement in accuracy is related to the exclusion of non-symmetrical polar-points (called bad points) to the emitter actual location.

It must be noticed that in Figure 6 the set of polar-points, adding a new receiver to the system, allows obtaining a region (ROI) more symmetrical to the emitter actual position. Accuracy in results improves by adding new receivers to the system. This filtered ROI is more symmetrical, both PPC and CHC (Algorithm 2) produce the same correct result.

**Algorithm 2.** CHC—Convex Hull Centroid Model Algorithm
**Data Input**
m>2 is the number of receivers. 
$C_{k}\left(x_{k},y_{k}\right)$, k=1,2,…,m, is the planar position of each receiver. 
$r_{k},k=1,2,\ldots ,m$, is the signal range of each receiver to an emitter.
**Procedure**
1: **Execute** the steps 1 until 4 of algorithm Polar Points Centroid Model.2: **Apply** (d-8), **find** the convex hull polygon for all polar-points in S. The obtained polygon is the minimal convex polygon that involves all interest points in S. This polygon constitutes the ROI.3: **Exclude** all polar-points on the boundary of this convex polygon, called bad polar-points, from S.4: **Apply** (d-9) in S**, compute** the location estimation, $E_{xy}\left(E_{x},E_{y}\right)$, of the emitter.
**Information Output**
5: Emitter location estimation $E_{xy}\left(E_{x},E_{y}\right)$.

**Figure 7:**
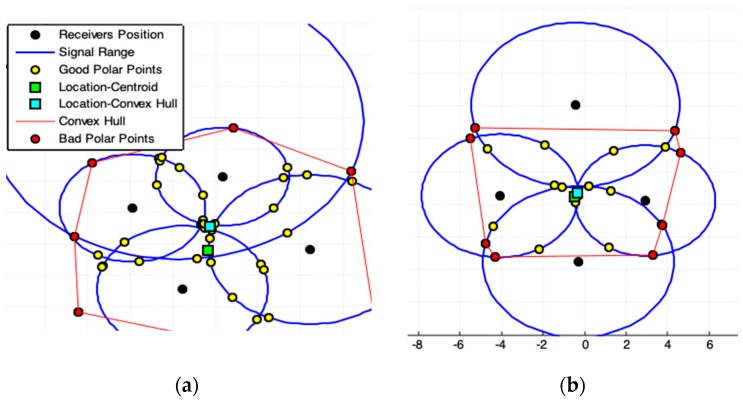
Different cases and respective responses of the PPC and CHC models. (**a**) Five receivers system. The data has some discrepancies. (**b**) Four receivers system. The data has small inconsistencies. For both cases, is better applying the solution provided by Section 2.1.

When the acquired data are coherent, the responses produced by the two models are similar (Figure 7b). If there are some discrepancies on the data, there may be divergences among the results (Figure 7a). Consequently, data errors promote differences in results. In this work, we do not evaluate data quality, but show consistency of results presented by the proposed models, whenever there is guarantee on the data quality.

### 2.4. Polar Lines Intersections Model (PLI)

The polar-line p is a line that passes through two corresponding polar-points $P{\left(x_{1},y_{1}\right)}^{*}$ and $Q{\left(x_{2},y_{2}\right)}^{*}$ that belongs to a line of a conic section (circle in this case). These polar-points are obtained by a given pole-point $E\left(x_{e},y_{e}\right)$ (Figure 1b). The PLI model (Algorithm 3) consists finding all combinations of pole-polar lines using as pole-points the position of each receiver at a time. After, we must compute the points of intersection among all these lines.

**Figure 8:**
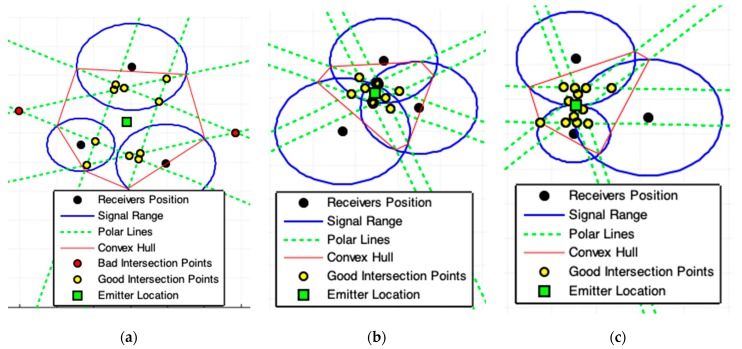
Results produced by Polar Lines Intersections Model (PLI). (**a**,**b**) show the possible presence of noise in acquired data. (**c**) Represents the best case for acquired data. For this case is better applying the exact solution provided by Section 2.1.

Figure 8a represents cases of data underestimation provide by sensors. Figure 8b shows a good case for real application. The emitter location should be on the common intersection point among the receiver range circles. Figure 8c shows the exact result. Reliable data quality improves accuracy.

**Figure 9:**
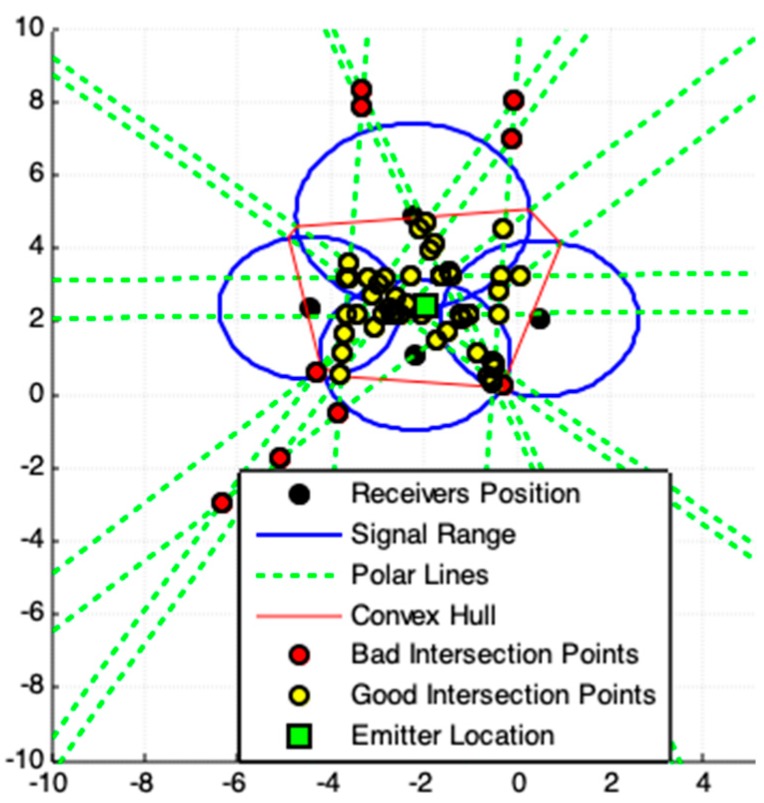
Result produced by PLI four receivers system. The non-symmetrical points are removed from the ROI by the use of Convex Hull. For this case is better applying the exact solution provided by Section 2.1.

A lot of pole-polar lines pairs, in the geometry arrangement with m receivers, are parallel lines (Figure 8 and Figure 9); this minimizes the number of intersection points. For m>3 receivers, several bad intersections points are generated. The convex hull among polar-points establishes a ROI that ensures better symmetry among the points of interest. For large values of m, many intersections will occur very far from the ROI, breaking then the desired symmetry among these points (Figure 9). The Pole-Polar Lines Intersections model achieves accurate values for emitter location because it generates significant symmetrical points of interest.

**Algorithm 3.** PLI—Polar Lines Intersections Model Algorithm
**Data Input**
m≥3 is the number of receivers. 
$C_{k}\left(x_{k},y_{k}\right)$, k=1,2,…,m, is the planar position of each receiver. 
$r_{k},k=1,2,\ldots ,m$, is the signal range of each receiver to an emitter.
**Procedure**
1: **for** each k=1,2,3,…,m
 2: ∀k,j:1,2,…,m/k≠j, by (d-6) and (d-7), for each receiver position $C_{k}$, used as pole-points, **compute** all combinations of polar-points $P_{kj}\left(x_{pkj},y_{pkj}\right)$ and $Q_{kj}\left(x_{qkj},y_{qkj}\right)$ with the respective receiver at position $C_{j}$ and signal range $r_{j}$.  3: **Stores** the corresponding points $P_{kj}$ and $Q_{kj}$ in the set R
$\left(R\leftarrow R\cup \left\{P_{kj},Q_{kj}\right\}\right)$. 4: For each corresponding $P_{kj}$ and $Q_{kj}$ points, by (d-6), **compute** the $p_{kj}$ polar-line equation.5: **end for**6: For all $p_{kj}$ polar-lines, by (d-3), **compute** the intersections points, $\left(x_{+},y_{+}\right)$, among all others polar-lines. **Stores** these intersections points in S.7: **Apply** (d-8), **find** the convex hull polygon for all polar-points in R. The obtained polygon is the minimal convex polygon that involves all interest points in R. This polygon constitutes our ROI.8: **Exclude** from S all intersections points among all polar-lines on the boundary, or out, of the ROI, called bad intersections points.9: **Apply** (d-9), **compute** the location estimation, $E_{xy}\left(E_{x},E_{y}\right)$, of the emitter.
**Information Output**
10: Emitter location estimation $E_{xy}\left(E_{x},E_{y}\right)$.

### 2.5. Tangent Lines Intersections Model (TLI)

**Figure 10:**
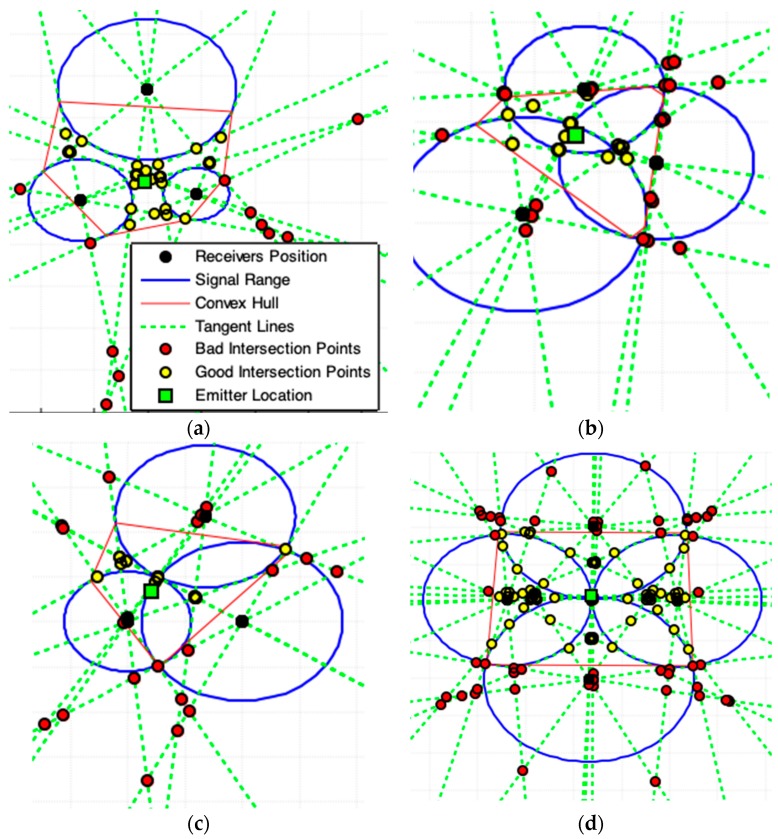
Results produced by Tangent Lines Intersections (TLI) model. (**a**,**b**) show the possible presence of noise in acquired data. (**c**,**d**) represents the best case for acquired data. (**d**) Use of four receivers (the legend box is omitted for best view). For cases (**c**,**d**) is better applying the exact solution provided by Section 2.1.

**Figure 11:**
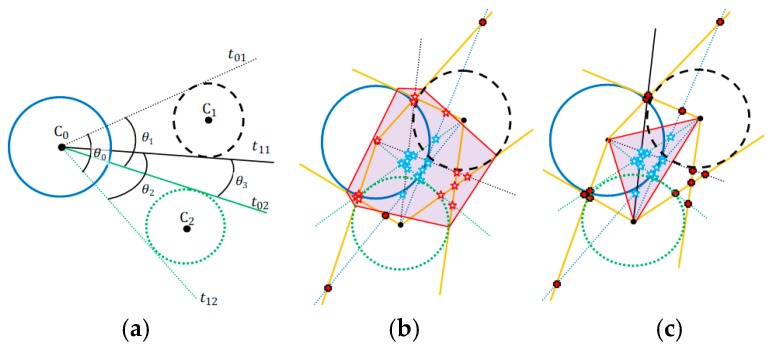
(**a**) Illustrations that define the Tangent Lines with Minimal Angles Model (MAI). (**b**) All intersections (red-star) with non-minimal angle tangent lines (orange-lines) must be eliminated. (**c**) All points inside the ROI formed by the convex hull polygon among the circle centers are the required points (blue-star).

Using each center circle point $C_{k}$ as pole-point, one by one, all polar-points $P_{kj}\left(x_{pkj},y_{pkj}\right)$ and $Q_{kj}\left(x_{qkj},y_{qkj}\right)$ can be computed for each other circle j≠k (Figure 1b, Figure 3 and Figure 10). It is possible to draw two lines for a particular $C_{k}$ point (tangent lines to a circle j≠k): one passing through by $P_{kj}$ and another by $Q_{kj}$ (Figure 10 and Figure 11a). If m is the number of receivers in the system (there are m circles), so, 2(m−1) tangent lines (Figure 10 and Figure 11a) pass through each circle and generate a large set of intersections points among these lines, but most of these points are bad points that are eliminated by the convex hull ROI. The convex hull among polar-points establishes a ROI that ensures better symmetry among the points of interest. For large values of m, many intersections will occur very far from the ROI, breaking the desired symmetry among these points.

There are intersections of tangent lines in each circle center because they pass by each circle center (receiver position) $C_{k}$. Whether these center points must be used in the estimation of emitter location depends on the geometric arrangement among receivers. While the results produced by TLI model (Algorithm 4) are consistent (Figure 10), m≥4 receiver usage is recommended (Figure 10d) for best accuracy.

**Algorithm 4.** TLI—Tangent Lines Intersections Model Algorithm
**Data Input**
m≥2 is the number of receivers. 
$C_{k}\left(x_{k},y_{k}\right)$, k=1,2,…,m, is the planar position of each receiver. 
$r_{k},k=1,2,\ldots ,m$, is the signal range of each receiver to an emitter.
**Procedure**
1: **for** each k=1,2,3,…,m
 2: ∀k,j:1,2,…,m/k≠j, by (d-6) and (d-7), for each receiver position $C_{k}$, used as pole-points, **compute** all combinations of polar-points $P_{kj}\left(x_{pkj},y_{pkj}\right)$ and $Q_{kj}\left(x_{qkj},y_{qkj}\right)$ with the respective receiver at position $C_{j}$ and signal range $r_{j}$.  3: **Stores** the points $P_{kj}$ and $Q_{kj}$ in the set R
$\left(R\leftarrow R\cup \left\{P_{kj},Q_{kj}\right\}\right)$. 4: For each corresponding $P_{kj}$ and $Q_{kj}$ polar-points, by (d-6), **computes** the respective two tangent lines equation $t_{Pkj}$ and $t_{Qkj}$ that passes by each $C_{k}$.5: **end for**6: For all $t_{Pkj}$ and $t_{Qkj}$ tangent-lines, by (d-3), **computes** the intersections points, $\left(x_{+},y_{+}\right)$, among all tangent-lines. Store these intersections points in S.7: **Apply** (d-8), **find** the convex hull polygon for all polar-points in R. The obtained polygon is the minimal convex polygon that involves all interest points in R. This polygon constitutes our ROI.8: **Exclude** from S all intersections points among all tangent-lines on the boundary, or out, of the ROI, called bad intersections points.9: **Apply** (d-9), **compute** the location estimation, $E_{xy}\left(E_{x},E_{y}\right)$, of the emitter.
**Information Output**
10: Emitter location estimation $E_{xy}\left(E_{x},E_{y}\right)$.

### 2.6. Tangent Lines with Minimal Angles Model (MAI)

A complementary explanation is necessary before presenting this model. We have to consider three disjoint circles centered at $C_{0}\left(x_{0},y_{0}\right)$, $C_{1}\left(x_{1},y_{1}\right)$, and $C_{2}\left(x_{2},y_{2}\right)$, as Figure 11a shows. Two tangent lines pass by $C_{0}$ through the circle at $C_{1}$ and two more through to the circle at $C_{2}$. Consider the angles $\theta_{k}$, k=0,1,2,3, formed between two tangent line pairs that touch the circles at $C_{1}$ and $C_{2}$. The tangent lines that touch the same circle are not important to this analysis. Discard all combinations of tangent lines pairs that touch the same circle. For the illustration presented by Figure 11a, four angles can be obtained by combining all possible pairs of tangent lines that touch different pairs of circles.

For each circle pair, the two tangent lines that form the lowest angle must be preserved, while others must be removed. These two preserved lines are used by MAI (Algorithm 5). Figure 11a shows that the two lines combinations with lowest angle are the tangents lines $t_{11}$ and $t_{02}$. Repeat this process to obtain all combinations of Tangent Lines with Minimal Angle that pass by different circles center to other m−1 circles for each different pair of circle. Executing this procedure, Figure 11b,c shows in orange-color the lines that must be eliminated.

**Figure 12:**
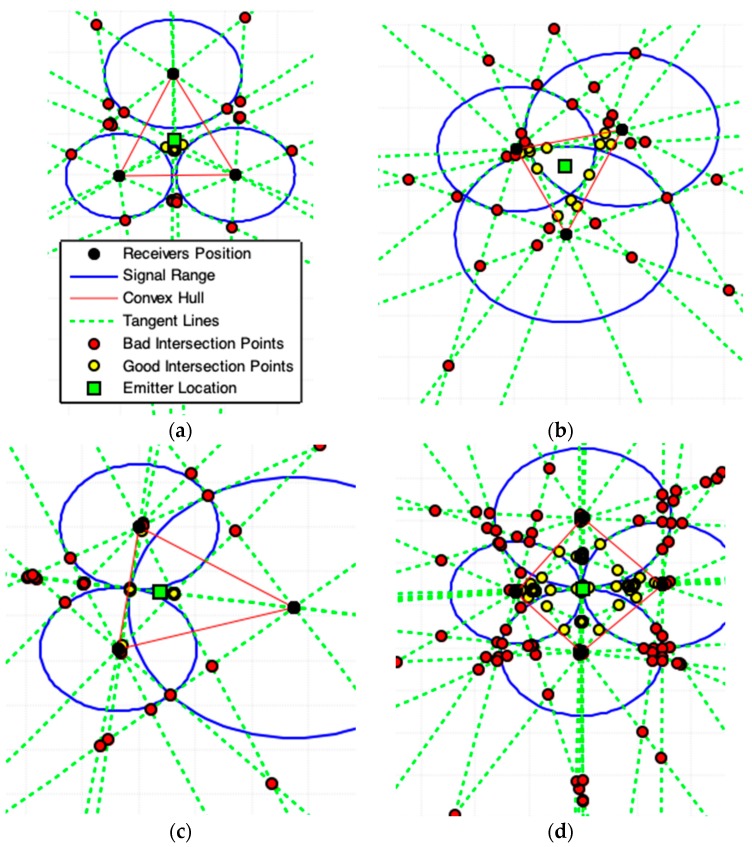
Results produced by MAI model. (**a**,**b**) show the possible presence of noise in acquired data. (**c**,**d**) represents the best case for acquired data. (**d**) Application using four receivers (the legend box is omitted for best view). For the cases (**c**,**d**) is better applying the exact solution provided by Section 2.1.

**Figure 13:**
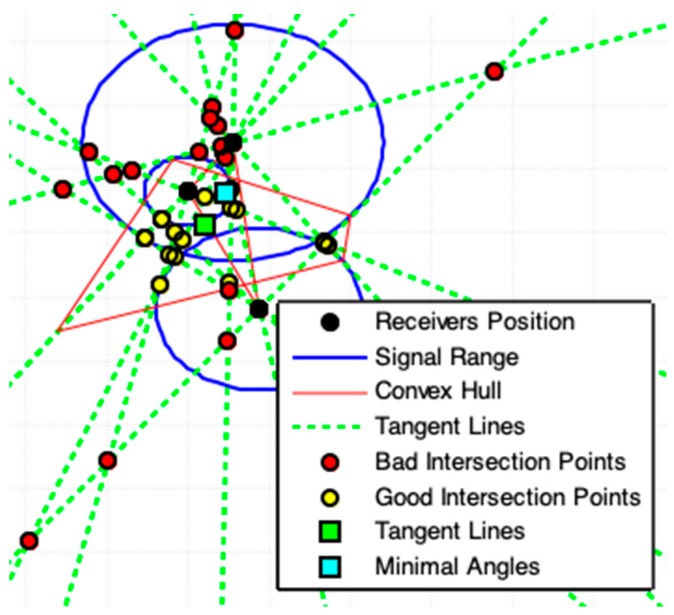
Geometric incoherence. The receiver is placed inside the range of another receiver. The geometric models TLI and MAI are able to overcome this data inconsistency and produce a satisfactory result.

Figure 12 and Figure 13 show some examples of results produced by applying the MAI. The results Figure 12a,b and Figure 13 are consistent with the expected results due to the signals range errors.

The signal range used in Figure 12c,d are accurate and the solutions are exact. But, for Figure 12c the MAI do not found this exact solution. MAI did not find the exact solution because the geometric arrangement of the devices of the localization system is not symmetrical. In case Figure 12d there is this geometric symmetry and the exact solution was found. However, it can be seen both in Figure 12c,d that there are polar points marking the exact solution and, if the exact solution exists, the best way to find it is to apply the solution provided in Section 2.1 of this work.

**Algorithm 5.** MAI—Tangent Lines with Minimal Angles Intersections Model Algorithm
**Data Input**
m≥2 is the number of receivers. 
$C_{k}\left(x_{k},y_{k}\right)$, k=1,2,…,m, is the planar position of each receiver. 
$r_{k},k=1,2,\ldots ,m$, is the signal range of each receiver to an emitter.
**Procedure**
1: **for** each k=1,2,3,…,m
 2: ∀k,j:1,2,…,m/k≠j, by (d-6) and (d-7), for each receiver position $C_{k}$, used as pole-points, **compute** all combinations of polar-points $P_{kj}\left(x_{pkj},y_{pkj}\right)$ and $Q_{kj}\left(x_{qkj},y_{qkj}\right)$ with the respective receiver at position $C_{j}$ and signal range $r_{j}$.  3: **Stores** the points $P_{kj}$ and $Q_{kj}$ in the set R
$\left(R\leftarrow R\cup \left\{P_{kj},Q_{kj}\right\}\right)$. 4: For each corresponding $P_{kj}$ and $Q_{kj}$ polar-points, by (d-6), **compute** the respective two tangent lines equation $t_{Pkj}$ and $t_{Qkj}$ that passes by $C_{k}$.5: **end for**6: For all $t_{Pkj}$ and $t_{Qkj}$ tangent-lines, by (d-3), **compute** the intersections points, $\left(x_{+},y_{+}\right)$, among all tangent-lines. **Stores** these intersections points in S.7: **Apply** (d-8), **find** the convex hull polygon for all center circles points $C_{k}$. The obtained polygon is the minimal convex polygon that involves all interest points in S. This polygon constitutes our ROI.8: **Exclude** from S all intersections points among all tangent-lines on the boundary, or out, of the ROI, called bad intersections points.9: **Apply** (d-9), **compute** the location estimation, $E_{xy}\left(E_{x},E_{y}\right)$, of the emitter.
**Information Output**
10: Emitter location estimation $E_{xy}\left(E_{x},E_{y}\right)$.

## 3. Experimental Cases 

### 3.1. Methodology Applied to Real Data Acquisition

**Figure 14:**
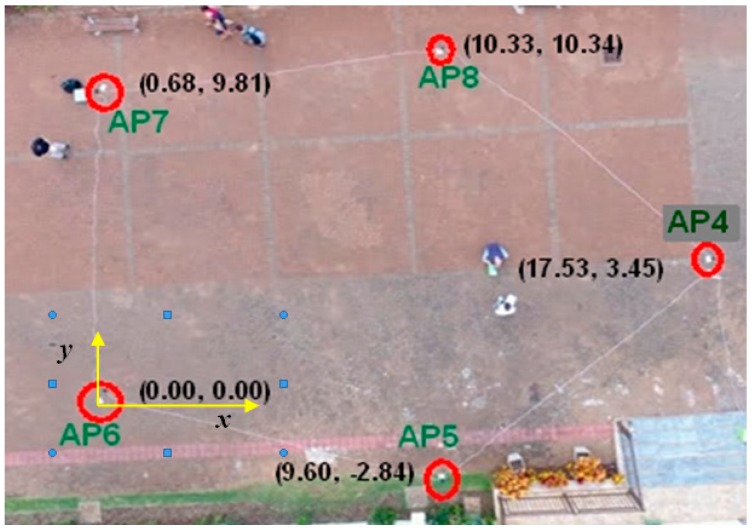
Structure composed of five Access Point (AP) devices and their respective position on an outdoor environment.

In order to carry out our experiments, a structure composed of five APs devices to compose the emitters system (AP4, AP5, AP6, AP7, and AP8) is set up in an outdoor environment that covers an area of 150 m^2^ on the ground (Figure 14). Each AP is positioned at the Cartesian coordinate (x,y) and the respective value (in meters) is registered alongside the respective AP (e.g., the AP5 is at (9.60,−2.84)—Figure 14). These Cartesian coordinate (2D) design the set of $C_{k}$ points (centers of range signals circles computed for the *kth*-AP) and defines the geometric arrangement of the covered area on the ground.

An AP is the interface between wireless and wired section of the network. Its task is supported by the IEEE 802.11 Network Infrastructures and mainly consists in a translation of the frames from wireless framing format into a wired network framing format like Ethernet. This experiment uses the AP Tp-Link Wireless N 300 Mbps TL-WR849N to compose the system of emitters that emits signals of radio frequency of 2.4 GHz. 

The receiver device is a commercial cell phone operating with the operational system Android. A previously developed application capable of receiving, identifying and measuring the strength of the signal sent by each emitter (APs of interest) was installed in this cell phone (our mobile device). 

To calibrate the mobile device it is placed at a distance of one meter away from each AP to acquire the respective signal strength of reference $\left(\rho_{0}^{k}\right)$ sent by the *kth*-AP.

Next, a set of coordinates $E_{p}$, marked and identified as known ground points, is located on the covered region. The mobile device is positioned on each of these marked ground points to receive the signals sent by each AP of interest. In this position, the strength of the signal $\rho_{k}\left(d\right)$ sent by the *kth*-AP is measured by the mobile device application.

**Figure 15:**
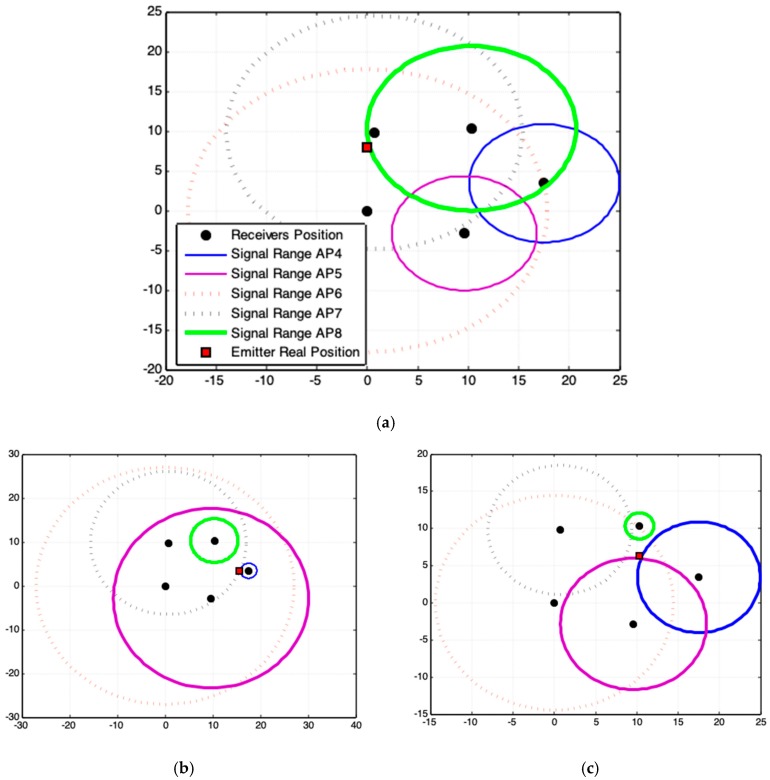
Acquired data quality (graphical units in meter). (**a**,**b**) worst quality data. (**c**) Satisfactory data quality.

By means of Equation (6), the radial signal-range $d=r_{kp}$ is computed for each *k*-AP located at $C_{k}$ using the signal strength $\rho_{kp}$ sent by the respective *k*-AP and received by the mobile device located at $E_{p}$ at each ground coordinate. The value ℒ=2.2 is adopted for the path-loss factor in order to use the Equation (6),

Thus, we know the position of each emitter k given by $C_{k}\left(x_{k},y_{k}\right)$; the radial signal-range at each AP given by $r_{kp}$ and; the real receiver position given by $E_{p}\left(x_{p},y_{p}\right)$ (Figure 15).

Now, through the proposed geometrical models, using $C_{k}\left(x_{k},y_{k}\right)$ and $r_{k}$ as input data, the location estimation $E_{xy}\left(E_{x},E_{y}\right)$ of the receiver can be computed and the distance between this emitter location approximation $E_{xy}$ to its known (real) position $E_{p}$ can be calculated.

The acquired data in this analysis cases are not preprocessed. Raw data are used to verify the behavior of the proposed geometric models in cases of works with data incoherency. 

### 3.2. Quality of Acquired Data

Figure 15 shows three sets of acquired data quality. Figure 15a,b are poor data quality and Figure 15c shows satisfactory data quality. Figure 15a shows that AP6 and AP7 overestimate and AP4 e AP5 underestimate the respective radial signal range. Although AP8 is more specific, the incoherence of data diverts the solution from the desired precision, independent of the method used to formulate the estimation. In light of this, [27] provided an alternative to improve the quality of the acquired data, [28] discussed the limits of localization using signal strength by a comparative study and [29] performed sensor localization under limited measurement capabilities. References [15,30] confirm that an optimal solution involves high computational cost, but obtaining the optimal solution is not guaranteed. This is because the errors are not in the methods that perform the triangulation of the signal data. The errors are in the signal data. 

With regard to the problem of localization in wireless sensor networks [31,32,33,34] presented alternative approaches and concerns regarding signal data behavior. To achieve better results, [34] preprocessed data after processing a set of partial results to decide which the best one was. As it can be noticed, acquiring accurate signal data is an arduous task. References [5,35] proposed methods for sampling signal data acquisition and discussed uncertainty and signal location. Besides, the environment is not predictable with multipath, fading, interference and shading effects [16].

In Figure 15a, AP6 generates a signal range that covers an area of ≈1200 m^2^ on the ground, while the area covered by the APs system is ≈150 m^2^ (Figure 14). Therefore, this data incoherence leads to solutions with larger errors.

### 3.3. Results and Analyses

Three experimental cases are chosen to show the results produced by the application of the proposed geometric models: (1) one that produces the worst result; (2) one that produces result with intermediate precision; and (3) one that generates more accurate result. The accuracy achieved in these cases is due solely to the quality of the data acquired. For more result, see Appendix A.

These analyses consider three metrics: (1) the error in distance measurement (Euclidean distance between the estimated location of the emitter $E_{xy}\left(E_{x},E_{y}\right)$ and the real emitter position $E_{p}\left(x_{p},y_{p}\right)$); (2) the error along *x*-axis given by $\left|E_{x}-x_{p}\right|$; and (3) the error along *y*-axis given by $\left|E_{y}-y_{p}\right|$.

The nomenclature used on the graphics legend box is:

- PPC—Polar Points Centroid Model (proposed).
- CHC—Convex Hull Centroid Model (proposed).
- PLI—Polar Lines Intersections Model (proposed).
- TLI—Tangent Lines Intersections Model (proposed).
- MAI—Tangent Lines with Minimal Angles Model (proposed).
- NRm—Newton–Rapson Method (for comparison).
- LSm—Least Square Method (for comparison).
- WLSm—Weighted Least Square Method (for comparison).

**Figure 16:**
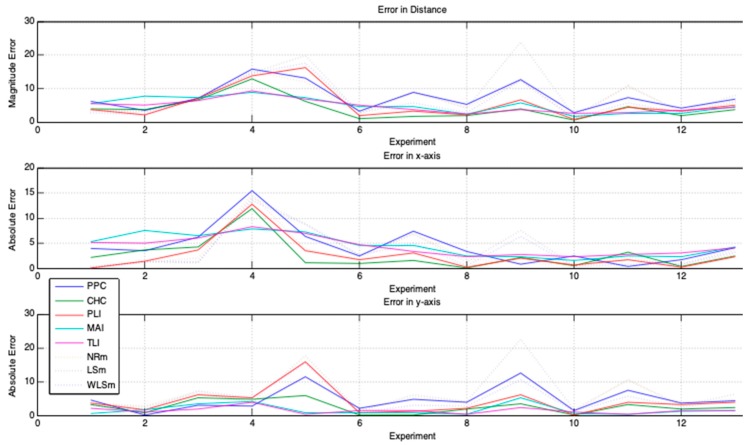
Magnitude error analyses.

For accomplishing the analyses, twelve experimental cases are generated (Figure 14) and the proposed geometric models (PPC, CHC, PLI, TLI, and MAI) are utilized in each acquired data set. We also use WLSm, NRm, and LSm methods to demonstrate the superior quality of the proposed geometric models. The metrics of analyses (distance error, *x*-axis error, and *y*-axis error) are computed for each result obtained from the geometric models and numerical methods. Figure 16 shows the magnitude of these errors for each experimental case. In these graphs, the 13th case represents the arithmetic mean of the magnitude errors for the respective metric and model/method.

Table 1 presents a global evaluation of the error values considering all experiments for each geometric model and numerical method. These values explain that some geometric model produce results with errors equivalent to those produced by numerical methods. MAI and TLI models promote larger errors on the *x*-axis, but smaller on the *y*-axis and distance (dist). They also promote smaller errors and produce smaller maximum errors, which show that MAI and TLI achieve better results when there is inconsistency in the data. CHC and PLI miss the least on the *x*-axis and get most significant result on the *y*-axis and distance. Errors committed by PPC are equivalent to those committed by numerical methods (WLSm, NRm, and LSm). We can observe that errors may be larger or smaller depending on the quality of the acquired data, but in the general context, geometric models present better results than numerical methods.

Table 2 displays a global mean error committed by the geometric models against numerical methods. That is to say, these values are the result of minimizing the errors reached by solving the problem of signal location. Errors are not committed by methods, but they are present in data, as Figure 15 displays. Table 2 shows that the proposed geometric models can achieve better results than those achieved by numerical methods.

Table 3 displays the variability of the errors committed by each method. The column Effective Variability of the Errors is the arithmetic mean of the standard deviation of the errors committed on the *x*-axis, *y*-axis and in distance by each method.

The Effective Variability of the Errors (Table 3) allows analyzing how a method is sensitive to the variation of data quality. In other words, this value is a metric to define how much a method can approach the exact solution when it operates on data with errors. The lower this value, the less sensitive is the method to the errors in the data and therefore has the ability to produce more accurate results. A simple analysis of the data in Table 3 shows that the proposed geometric models are more robust when they operate on data with errors than the traditional WLSm, NRm, and LSm methods. Among the geometric methods, the TLI has greater capacity to process data with errors.

**Figure 17:**
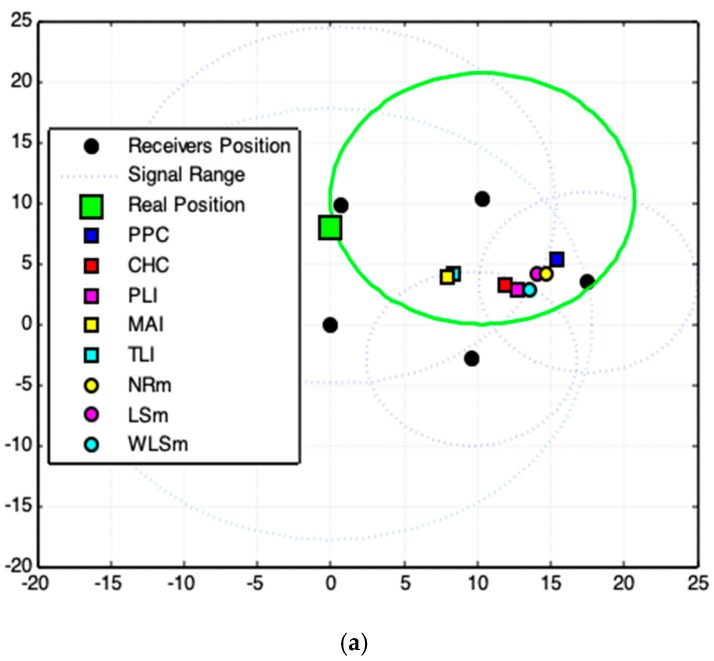

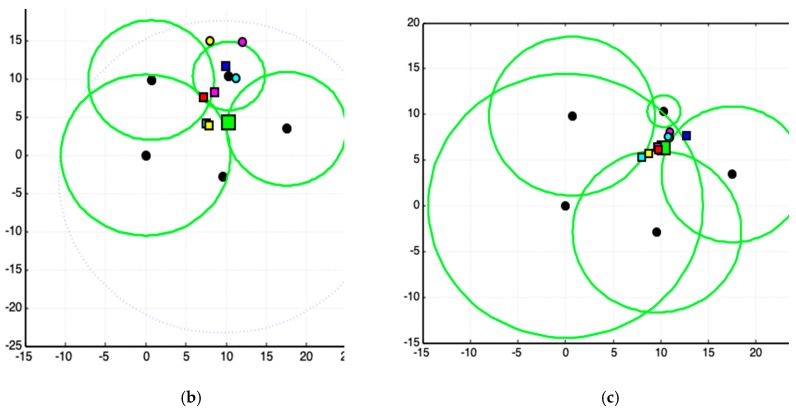
Some particular experimental results. (**a**) The worst case. (**b**) An intermediate case. (**c**) The best case. The blue-dotted circle lines show the receivers/emitters that suffered severe interferences and promoted the acquisition of data with errors.

In order to analyze these results in a particular context, Figure 15 prove that the fourth experiment is the worst case of acquired signal data, since it promotes bigger errors, both for the numerical methods and for the geometric models (Figure 17a). An intermediate result is achieved by the eleventh (Figure 17b) experiment and the best result is obtained in tenth (Figure 17c).

Figure 17c shows that when satisfactory data make all methods and models works with relative precision, whereas data incoherencies (dotted circle lines in Figure 17) make results divert of the expected solution, and Figure 17a–c confirms that the proposed geometric models point to a location closer to the exact position of the signal source than the numerical methods used and can better solve the existing problem of data inaccuracy.

The worst case, Figure 17a, deals with the case of inconsistency of acquired data. Even, if the use of such data does not show the precise location of the emitter, the results produced by the geometric models are consistent with the geometric arrangement among data. The solution is found on the region where most of signals range (circles) intersects. The solution is inaccurate because of the serious deviations in the acquired data. If data inconsistency is moderate (Figure 17b), the proposed geometric models generate higher quality results than those produced by numerical methods.

The Appendix A presents six other experimental results.

## 4. Conclusions

Our results show that there is a geometrical relationship between the emitter location and elements of pole-polar geometry. For this aim, we propose geometric models to solve a geometric problem: 2D location of a signal source and, if accurate data are available, an exact solution is found with cost O(n) (Section 2.1). If the data quality is satisfactory, the proposed models obtain the best estimative for the problem of locating the signal emitter source and are more robust when they operate on data with errors than the traditional WLSm, NRm, and LSm methods. 

This work contributes to cope with the problem of applying 2D point location in a geometric relationship by reducing the number of arithmetic operations needed by the current conventional methods in use and the inherent propagation errors in the acquired data. The proposed geometric models have low computational cost O(nlogn). The obtained results are consistent even when there is incoherence in the data or when there is incoherence geometric in the arrangement of the receivers system.

When analyzing these results, we observe that the solutions given by the geometric models are located around the region of intersections of the radial signals ranges for all cases. Thus, a linear combination of all these solutions is also a solution. Consequently, we have computed the mean among the five solutions to derive this linear combination with no special motivation. This new solution minimizes the global error (Table 2).

Five geometric models have been presented and experimented with displayed solutions that are located around the region of intersection of the radial signals range for all cases. A comparison of results with numerical methods Weighted Least-Square, Least-Square, and Newton–Rapson has been carried out and results achieved by the proposed geometric models have improved, especially in cases of moderate data incoherence.

The proposed geometric models have the following limitations: (1) 2D solution (we are developing a 3D solution). (2) Proposed methods for estimating location require that the device to be located be inserted into the coverage area of the APs. We are developing a solution to this limitation in conjunction with the 3D solution. (3) APs may be arranged collinearly only for a few specific cases.

To develop this work we have built a simulator in MatLab code and used the implemented functions to construct a prototype that processes real data [36]. All code and data used are at anyone’s disposal. Go to www.coding2change.org/articles/GeometricModels.zip. Consult User’s Manual for more instructions.

## Figures and Tables

**Table 1:** Magnitude of errors in the experimental cases.

Methods	Magnitudes Errors (in Meters)								
	Minimum Error			Maximum Error			Mean Error		
	*x*-axis	*y*-axis	Distance	*x*-axis	*y*-axis	Distance	*x*-axis	*y*-axis	Distance
PPC *	0.4	0.2	2.7	15.5	12.5	15.7	4.2	4.4	6.9
CHC *	0.1	0.1	0.6	11.9	6.0	12.8	2.5	2.4	3.7
PLI *	0.1	0.1	0.7	12.8	15.9	16.3	2.4	3.8	5.0
MAI *	1.6	0.3	1.7	7.9	5.3	8.9	4.2	1.5	4.7
TLI *	2.3	0.3	2.3	8.3	3.9	9.2	4.1	1.3	4.4
NRm	0.1	1.1	1.3	14.7	12.2	15.2	2.8	5.1	6.5
LSm	0.3	1.2	1.8	14.1	22.5	23.7	3.6	6.4	7.9
WLSm	0.1	0.7	1.3	13.6	15.3	17.7	3.5	4.9	6.5

* Geometric Models proposed in this work.

**Table 2:** Global mean errors (Geometric Models × Numerical Methods).

	Mean Errors (in Meters)		
	*x*-axis	*y*-axis	Distance
Geometric Models	3.7	2.9	5.3
NRm + LSm+ WLSm	3.5	4.1	6.0

**Table 3:** Standard deviation of the errors.

Methods	Standard Deviation of the Errors			Effective Variabilityof the Errors
	*x*-axis	*y*-axis	Distance	
PPC *	4.1	3.8	4.5	4.1
CHC *	3.1	2.1	3.4	2.9
PLI *	3.3	4.1	4.9	4.1
MAI *	2.5	1.6	2.6	2.2
TLI *	2.3	1.0	2.3	1.9
NRm	4.0	4.0	4.9	4.3
LSm	4.3	6.7	7.4	6.1
WLSm	4.2	4.3	5.5	4.7

* Geometric Models proposed in this work.

**Figure A1:**
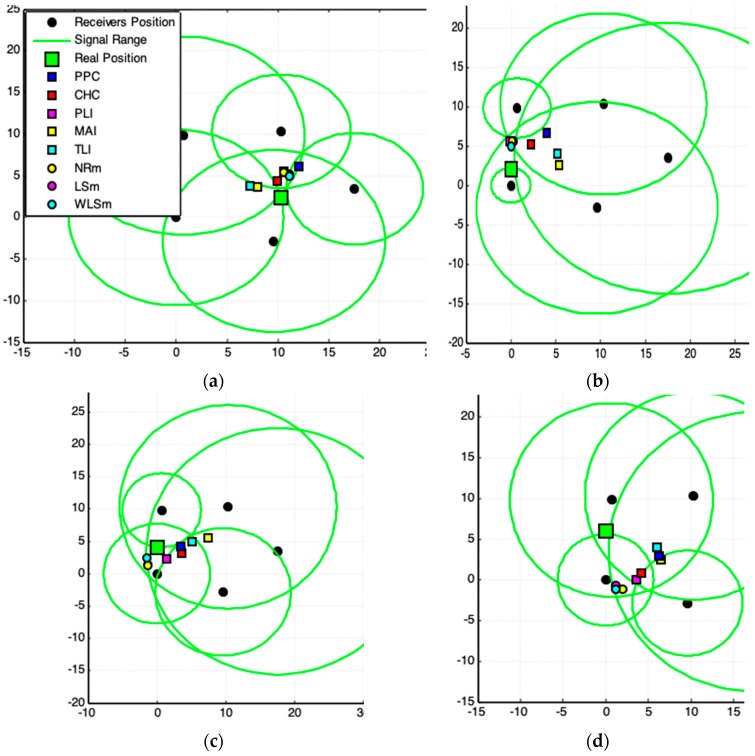

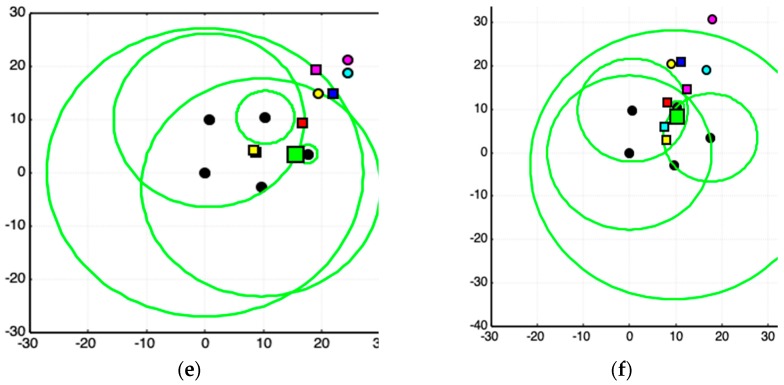
Complementary experimental results.
